# Meteorological Table

**Published:** 1811-08

**Authors:** 


					['175 ]
METEOROLOGICAL TABLE.
From June 27, to July ~7.
^ I Therm. | Barom.
12?
,28
!2g
bo
i
0
Winds. ]Atmo. Variations,
b3
|65
55
63
53
66
33
35
56
60
62
157
963
C^il2'
|l3lS4
63
S
67 65 29s
68 66 29?
67 66
66 65
67 66
72 68
61 56
59 57
66 58
69 6*
66 6d
70 60
70 6/.
77 65
77 66
80 67
114
l?
:>*
33
33
35
68
53
56
59
58
Jei
26 63
,27164
75 66 299
65 63
70 65
70 62
72 64
72 68
74 64'297
? 59299 30
? 55130
69 61,299 301
67 63 30
v t quantity of rain from .Tune 27, to July 27, S inches T2,^cT. Tbe pre-
1 lng wind has been N. E. from which ic has blown 12 days, and very fre-
of ?'y ur't^1 a clouded or h izy atmosphere, particularly on the 29th and 30th
'rh T anC^ 00 t^e *st> ^ Ju^- l^th, Thunder. Though the
tirn'*was, with the exception of the 2d, always below 70 on these hazy
^p' 5he quality of the heat w^.s unusually oppressive.
Prices Street, Cavendish. Square

				

